# Editorial: ‘Inter-identities' in Life, Mind, and Society

**DOI:** 10.3389/fpsyg.2021.704772

**Published:** 2021-06-18

**Authors:** Arantza Etxeberria, Luisa Damiano, Kepa Ruiz-Mirazo

**Affiliations:** ^1^Department of Philosophy, IAS Research Centre for Life, Mind, and Society, University of the Basque Country (UPV/EHU), Donostia-San Sebastián, Spain; ^2^Department of Communication, Arts and Media, IULM University, Milan, Italy; ^3^Biofisika Institute (CSIC, UPV/EHU)—Basque Centre for Biophysics, Leioa, Spain

**Keywords:** complexity, individuality, interaction, relational autonomy, agency

The complexity of life, mind and society seems to be an endless source of challenges for philosophy and science, which keep exploring them under ever-new lenses. In order to explain or describe these domains, systemic approaches investigate how parts are dynamically organized and integrated, *vertically*, as wholes articulated in many levels, whereas interactive views delve into properties, entities and processes characterizing *horizontal* relations. Here complexity, though grounded in features that are conceived as individual and cohesive, is most prominently recognized to arise from interactions at the intersections among systems forming wider ensembles or diverse consortia. On this basis, individuality and identity of wholes are understood as affected and/or constituted by interactive dynamics with other systems and the environment.

Classical approaches often comprise individualistic assumptions that neglect interactions and collective phenomena related to identity. Naturalist accounts have to confront significant trouble in conceptualizing identity and individuality, since the massive interconnectivity and/or heterogeneity among components intrinsic to complex organizations entail remarkable difficulties in determining primitive systems or starting points. Currently, new modeling and explanatory approaches engage in studying a variety of processes, such as metabolic interactions giving rise to the first living cells, their evolution through lateral transfer of genes, the emergence of multicellular organisms, the intersubjective participation in sense-making or in the generation of cognitive meaning, social interplay as a source of autonomous decision-making, and technologically mediated interrelation in social groups and network dynamics.

Rather than focusing on substantial identities, these endeavors examine how the entities involved in those processes appear, change and act *in context*. This perspective excludes the possibility of conceiving such entities as independent individuals, and requires to consider them as entangled. When defining the title of this Research Topic, we coined the term “inter-identities” to refer to these kinds of interactions and, more precisely, to indicate what emerges from these interactive processes and cannot be found in the interactors, when taken separately. We introduced the notion of *inter-identities* with a programmatic aim: promoting the exploration of new strategies to study and conceptualize these forms of complex interaction.

The articles gathered in this Research Topic (RT) propose inquiries convergent to that goal. They are based on multiple transdisciplinary approaches aiming at describing complex interactive processes belonging to the living, the cognitive and the social domains.

A central feature of the systems populating these three domains, typically captured through the notion of autonomy, is the capability of coupling with the environment and other systems. An autonomous system cannot be interpreted as fully determined by internal, localized elements, like genes, nor by external factors. The property of autonomy entails systems whose dynamics of self-production are based on interactions between the systems as wholes, their components and their environments. Hence, these systems' autonomous behavior is deeply grounded in interactive dynamics, so that the autonomy involves normative assessments of the environment, in such a way that other systems and situations are perceived and cognized according to those interactive norms, as well in normative constraints prompted from the environment.

In this sense, for living, cognitive and social systems the notion of *autonomy* and the notion of *inter-identity* are deeply interconnected: they actually articulate a tension between, on one side, the self-determined character of these systems' dynamics of self-production and generation of behavior, and, on the other side, the multiple connections influencing these processes.

This tension travels across the articles contributing to the RT. The related problems concern different scientific and philosophical aspects in the three target domains of life, mind and society, or more transversal, epistemological dimensions of inter-identities, and the research challenges they involve.

(a) Life and Biology. An important fraction of the articles engages with inter-identities in this realm. In the field of the *origins of life*, Ruiz-Mirazo et al., suggest that the first living cells already comprise an irreducible collective dimension, both in ecological and evolutionary terms, necessary to explain how several bottlenecks of the process of biogenesis are overcome. About the organization of the motility of *eukaryote cells* in interaction with the environment, Militello argues that its emergence involves a strict control of the motile abilities of their constituting entities, such as organelles of endosymbiotic origin (i.e., mitochondria and plastids) and flagella, although it does not necessarily entail a complete loss of the agential capacities of the individual parts. Expanding the discussion toward other *complex collectivities*, Canciani et al., build an organizational account of eusociality which emphasizes the regulatory control relationships involved in some insect colonies (e.g., *Apis mellifera*, their case study), involving a hierarchically organized network of interactions.

Looking at examples in *synthetic biology* Bich takes a complementary view on the problem of “inter-identity” by studying the ways in which models and realizations of that field can contribute to discover the interactive dimension of minimal life and cognition. In particular, the article discusses how concepts such as control, cognition, communication can characterize those interactions.

Other papers explore *evolutionary processes* for which interactions are decisive. Hernández and Vecchi investigate how biological autonomy is compatible with evolutionary processes by which living organisms capture and integrate environmental ingredients directly within their own dynamic organization; they analyse, in particular, *biotic entrenchment* emphasizing the interactive aspects of the construction of the individual. Also within this line, an illustrative case of the evolutionary relevance of interactions of interspecific individuals is offered by Suarez and Triviño on the sanguivory diet of vampire bats as an adaptation occurring at the level of the holobiont, the *host–microbiome multispecies* systems. Besides, Nuño de la Rosa et al. consider the evolution of eutherian pregnancy as the relational emergence of the pregnant female a new kind of *historical individual*, in contrast with prevalent dualistic conceptions often based on conflict models about the relationship between mothers and embryos.

(b) Minds and psychology. A second group of articles examines issues related to cognitive agency and identity in interactions.

On personal identity in social interaction from an enactive standpoint, James and Loaiza characterize inter-personal identities as collections of entangled stabilities that emerge in recurrent social interaction, and emphasize how sense-making, at the personal level, is a manifestation of histories of acting together. Another focus of interest is the role of social interactions in non-heterosexual personal identity; Collado and Besoain introduce a processual theory to examine the suffering associated with the influence of prevalent heteronormative environments on identities. In order to overcome standard monologic accounts, they consider how multiple effects or self-states can be produced, recurrently unified to create identities inherently susceptible to transformation, for example through performance art. Addressing pregnancy from an agential perspective, Martínez Quintero and De Jaegher converge in prospecting the interactive developmental organization of mother-baby relations, and stress the importance of taking into account the two inter-connected agencies, as well as how maternal agency changes while pregnancy advances.

(c) Societies, education, bioethics. This group of articles offers specific and insightful examples of inter-identities. Inter-identities in *education* is the topic explored by Pérez-Izaguirre in the context of a Basque secondary school in which adolescent students, coming from diverse ethnic backgrounds, meet and tend to construct their identity through transgression. Arrieta's article concerns the domain of *bioethics* in medicine. Based on the discussion of patients' decisional, executive, and narrative capacities, the author proposes a characterization of the patient's autonomy in the case of poor treatment adherence, and examines what appropriate actions may contribute to increase adherence rates. The collective dimension of *social and political* forms of organization is explored by Barandiaran et al., within the framework of networked digital interactions. By means of a detailed and critical exploration of different theoretical views of identity, they characterize collective identities as recurrent, cohesive, and coordinated communicative interaction networks, following a technical graph–theoretical approach.

(d) *Epistemic dimensions of interidentities*. Although all the articles contribute to the study of interidentities, some of them are particularly dedicated to more general epistemic matters. Thus, Gómez-Marin and Arnau examine the *epistemological* changes required to conceptualize *inter-identities*, which should be rather thought, according to their Whiteheadian (internalist-relational) account, as “*intra-identities.”* They criticize not only reductionist, but also emergentist positions, arguing in favor of a general shift of perspective toward process ontology to overcome difficulties attributed to the *-simple location-* assumption, which gives the false impression that enduring substances exist. Through the analysis of some experimental and modeling practices of biologists, Montevil and Mossio explore the way in which historical and relational approaches interpret the identity of organisms. The acknowledgment of a complementary relation between these views leads the authors to promote a compelling convergence of these perspectives in a hybrid construct. Also within philosophy of biology, Ferreira-Ruiz and Umerez discuss interactionism with regard to gene-centrism and the nature-nurture problem. They criticize the vagueness that general interactionist accounts tend to show, and propose examining more carefully the causation behind complexity in order to clarify the interactionist claims (e.g., causal parity) supporting deflationary positions with respect to genetics.

In general, the contributions gathered in this RT tackle, from different perspectives, two main sides of a common issue: how do complex (biological, cognitive, social) systems construct their identities? And also: how do science and philosophy conceptualize and methodologically explore interactivities? We believe that the answers they propose constitute a remarkably diverse and thought-provoking body of research, delineating new approaches to describe the processes of interaction in which complex identities—*inter-identities*—emerge.

## Author Contributions

All authors listed have made a substantial, direct and intellectual contribution to the work, and approved it for publication.

## Conflict of Interest

The authors declare that the research was conducted in the absence of any commercial or financial relationships that could be construed as a potential conflict of interest.

